# Modifying Microstructure and Improving Mechanical Properties of New Ti-Al-V Titanium Alloys via Fe Addition

**DOI:** 10.3390/ma17215161

**Published:** 2024-10-23

**Authors:** Guoqing Dai, Hai Gu, Jie Zhang, Jie Jiang, Jianhua Sun, Zhonggang Sun

**Affiliations:** 1 School of Mechanical Engineering, Nantong Institute of Technology, Nantong 226001, China; guoqingdai@njtech.edu.cn (G.D.); zhangjie@ntit.edu.cn (J.Z.); jiangjie@ntit.edu.cn (J.J.); sjhuant@ntit.edu.cn (J.S.);; 2 Jiangsu Key Laboratory of 3D Printing Equipment and Application Technology, Nantong Institute of Technology, Nantong 226002, China

**Keywords:** titanium alloys, fe addition, nanoindentation, microstructure refinement, slip system activity

## Abstract

A comprehensive study was carried out to investigate the effects of Fe addition (0–0.9 wt.%) on the microstructure evolution and mechanical properties of Ti-6Al-4V alloys. The results indicate that Fe addition has a significant refinement effect on the microstructure of titanium alloys; specifically, 0.9 wt.% Fe addition can lead to a 47.37% decrease in the width of lamellar α. The modulus also decreases by 18.89% with the increase in the Fe content, being 91.40 GPa in Ti-6Al-4V-0.9Fe. And the microhardness and wear resistance are improved due to Fe addition. In addition, the constitutive equation of the Fe content and the elastic compliance coefficient were calculated, which can better describe the relationship between Fe addition and the elastic–plastic properties of titanium alloys. The slip systems’ activity during the deformation process was also discussed using the Schmid factor. It shows that Fe addition is beneficial for the activity of prismatic and pyramidal slip systems, especially in the {10$\bar{1}$0} <11$\bar{2}$0>, {10$\bar{1}$1} <11$\bar{2}$3>, and {11$\bar{2}$2} <11$\bar{2}$3> slip systems.

## 1. Introduction

Ti-6Al-4V has been extensively utilized across diverse industries, including in aerospace, marine, construction, and biomedical devices [1,2,3]. This widespread adoption is primarily attributed to its remarkable mechanical properties and exceptional corrosion resistance [4,5]. However, the broader application of Ti-6Al-4V is limited by its deformation behavior and workability at room temperature. Specifically, improvements in the elasticity and plasticity of Ti-6Al-4V are necessary to meet evolving demands. Consequently, numerous efforts have been undertaken to enhance the deformation properties of Ti-6Al-4V, employing various approaches such as heat treatments [6,7], isothermal forging [8,9], and microalloying [10,11]. Among these methods, microalloying has proven to be a straightforward and effective approach to enhance alloy properties, particularly with the addition of β-stabilizing elements such as B, Cu, Cr, and Fe. Zhang et al. [12] found that the incorporation of Cu not only refines the grain structure but also enhances the material’s strength. Niu et al. [13] discussed the formation of a Ti-B interstitial solid solution resulting from the addition of B, which serves as a nucleation point, thereby facilitating the development of finer grains. However, it is important to note that the addition of both B and Cu may negatively impact the plasticity and elongation of titanium alloys, highlighting the need to consider alternative elements for microalloying to improve these properties. Korneva et al. [14] investigated the effects of high-pressure torsion (HPT) deformation on Ti-Nb and Ti-Mo alloys and found that the thermal stability of the ω phase is influenced by the concentration of alloying elements present in the phase. Specifically, an increase in the content of these alloying elements correlates with enhanced thermal stability of the ω phase. Furthermore, the incorporation of β-stabilizing elements, such as Nb, Mo, Fe, and Co, can significantly enhance the thermal stability of the ω phase. Gornakova et al. [15] conducted a study on titanium–iron binary alloys and discovered HPT resulted in the formation of a metastable high-pressure ω-Ti phase, which persisted even after the pressure was released. Straumal et al. [16] investigated the structure and microhardness of a Ti-4 wt.% Co alloy following pre-annealing and high-pressure torsion. Their findings indicate that HPT leads to the transformation of α-Ti into ω-Ti. Notably, as the pre-annealing temperature rises, the proportion of the ω phase diminishes, decreasing from the range of 60–65% at 500 °C to approximately 5% at 600 °C. Additionally, the microhardness of all examined samples increased with higher pre-annealing temperatures. Kilmametov et al. [17] found that high-pressure torsion of the Ti-Co alloy results in α to ω and β- to ω phase transformations. MacLeod et al. [18] found that Ti-6Al-4V remains stable in the hexagonal close-packed phase or α phase up to temperatures of approximately 30 GPa and 886 K. The effects of temperature on the volume expansion and compressibility of α-Ti-6Al-4V are minimal. The martensitic α → ω (hexagonal) transformation occurs at around 30 GPa, with both phases coexisting until the transition to the ω phase is completed at approximately 38–40 GPa. Between 300 K and 844 K, the α → ω transition appears to occur independent of temperature. The ω-Ti-6Al-4V phase is stable at approximately 91 GPa and 844 K. Smith et al. [19] investigated the room temperature compression and equation of state of a series of titanium (Ti) alloys stabilized in the body-centered cubic (β) phase, which demonstrated good phase stability within the studied pressure range. Only the Ti-36Nb-2Ta-0.3O alloy exhibited a second phase, and this occurred only at extremely high compression levels. While the presence of oxygen can significantly influence the relative stability of phases in pure titanium under pressure, it was found to have a minimal effect on the ability of molybdenum (Mo) to stabilize the β-phase titanium and had little impact on the elastic properties of the Ti-Mo binary alloy. The observed effects are surprisingly small. Errandonea et al. [20] found that uniaxial stress decreases the stress levels at which transitions are both initiated and completed.

Previous reports indicate that Fe demonstrates a remarkable ability to significantly stabilize the β-phase, and its addition to titanium alloys substantially enhances the overall properties. The microstructure evolution and mechanical properties of Ti-1Fe and Ti-3Fe alloys were investigated by Sandlöbes et al. [21]. The inclusion of Fe resulted in enhanced ultimate tensile strength and hardness in these alloys. Similarly, Niu et al. [22] observed elevated strength and a positive strengthening effect, along with favorable plasticity and modulus, in Ti-Fe alloys, surpassing other titanium alloys. These findings substantiate the substantial influence of Fe on the properties of titanium alloys. Chen et al. [23] found that the comprehensive mechanical properties of Ti6Al4V-0.55Fe alloys are superior than those of Ti-6Al-4V alloys. The elongation also increased without any significant decrease in the yield strength or ultimate strength. Liao et al. [24] discussed the optimal strength, plasticity, and toughness to exhibit the effect of Fe addition in several conditions. Rabadia et al. [25] pointed out that Fe is a low-cost and strong β-stabilizing element in titanium alloys, which also reduces the precipitation of the α phase and reduces the start temperature of martensite, thus facilitating the retention of the β-phase at room temperature. Therefore, it is beneficial for the tensile strength and bending strength.

These results display that titanium has better comprehensive mechanical properties with Fe addition, but few studies were conducted to determine the nanomechanical properties of Ti-6Al-4V-*x*Fe alloys, which actually need to be known to better understand the influence of Fe addition. Nanoindentation is an effective technique to obtain the elastic modulus, hardness, and plasticity mechanisms in materials science, and it can be used to determine the nanomechanical behavior of different alloys [22,23,24,25,26,27,28,29]. Although the actual deformation process of nanoindentation is simple, the matrix materials and kinematics involved are complex. And the different microstructures can be compared by observing the structure formations below and around the indentations. According to previous studies, the material around the contact area is deformed in different shapes and accompanied by phenomena such as pile-up and sink-in, and these features are directly related to the material composition [30,31,32].

This study addresses the problems of low thermo plasticity, high deformation resistance, and non-uniformity of organization that exist in the current hot working process of titanium alloys. With the purpose of reducing the deformation resistance of titanium alloys and improving the hot working performance of titanium alloys, we make use of the characteristics of the iron element to reduce the rheological stress of titanium alloys, and we use microalloying with trace amounts of the iron element as a new means to improve the hot working process of titanium alloys. The aim of this work was to investigate the effect of Fe addition on the modulus and plasticity properties of Ti-6Al-4V. The microstructure characterization and nanomechanical properties of Ti-6Al-4V-*x*Fe (*x* = 0–0.9 wt.%) alloys was examined, and the equation to determine the relation between the Fe content and the elastic compliance constants was calculated. The relation between the Fe content and slip system activity was also discussed to explain the improvement in mechanical properties.

## 2. Materials and Experiments

### 2.1. Materials and Microstructure Characterization

New Ti-6Al-4V-*x*Fe (*x* = 0–0.9 wt.%) alloys were designed and fabricated from sponge titanium, Al-V master alloy, and pure iron (99.95%). An amount of 1 kg of cast samples were made by vacuum suspension induction melting with a water-cooled copper crucible at 1700 °C under the protection of argon. The melting process involved flipping and re-melting six times for homogeneous composition distribution. The specific element content was determined by coupled plasma atomic emission spectroscopy (Agilent 5110, Santa Clara, CA, USA).

These Ti-6Al-4V-*x*Fe alloys were annealed at 1050 °C for 30 min and at 700 °C for 2 h and cooled in furnace to eliminate the initial stress. The samples for microstructural observation were grinded and polished mechanically and chemical etched with Kroll’s reagent (10 vol% HF, 20 vol% HNO_3_, 70 vol% H_2_O). The microstructures were observed by optical microscopy (OM, Zeiss Observer A1m, Zeiss, Oberkochen, Germany), scanning electron microscopy (SEM, Hitachi SU 70, Hitachi, Tokyo, Japan), and electron backscatter diffraction (EBSD, Oxford Instruments, Oxfordshire, England). Phase analysis was performed by X-ray diffraction (XRD, D8ADVANCE, Cu Kα, Cambridge, MA, USA) at a scanning speed of 4°/min. And the samples for nanoindentation were electro-polished with a solution of 5% perchloric acid in alcohol under constant voltage (32 V) for 180 s at 25 °C. Surface characterization of the specimens was performed using a Nanosurf Easyscan 2 (Nanosurf, Liestal, Switzerland) atomic force microscope. The cantilever utilized in this study was of the EZ2 head type, featuring an aluminum reflecting coating and equipped with a contact mode AFM probe.

### 2.2. Nanoindentation Test

The nanoindentation tests were carried out using an G200 indenter (Agilent, Santa Clara, CA, USA) with a Berkovich diamond indenter, and the indenter load was 30 mN with the same loading rate at ambient temperature (25 °C) under the laboratory environment. In addition, 15 indents were repeated for each nanoindentation condition, and the average values were used during this study. Furthermore, different penetration depths and applied loads were utilized to analyze the micro-mechanical behavior of Ti-6Al-4V-*x*Fe alloys.

The Oliver and Pharr method was applied during this study, and the hardness and Young’s modulus can be obtained from the maximum load and the initial unloading slope using this method [33,34]:

(1) $$H=\frac{P_{max}}{A_{c}}$$

where *H* is the hardness at the ratio of the maximum applied load *P_max_*, and *A_c_* is the contact area of the indentation.

(2) $$A_{C}=C_{0}{h_{c}}^{2}+C_{1}{h_{c}}^{1}{+C}_{2}{h_{c}}^{1/2}{+C}_{3}{h_{c}}^{1/4}{+\cdots +C}_{8}{h_{c}}^{1/128}$$

where *K* is a constant, and the value is 24.56 for a Berkovich indenter. And *h_c_* is the contact depth at the maximum load, which can be estimated as follows:

(3) $$h_{c}=h_{max}-\varepsilon \frac{P_{max}}{S}$$

where *h_max_* is the displacement at the maximum load, ε is the constant related to the indenter tip geometry and has a value of 0.72 for a conical indenter, 0.75 for a Berkovich indenter, and 1 for a flat cylindrical indenter. In addition, ***S*** is the elastic contact stiffness and can be calculated by unloading slope at maximum indentation depth *h_max_* as follows:

(4) $$S={\left(\frac{dP}{dh}\right)}_{h=h_{max}}=\beta \frac{2}{\sqrt{\pi }}E_{r}\sqrt{A_{c}}$$

(5) $$\frac{1}{E_{r}}=\frac{1-v_{s}^{2}}{E_{s}}+\frac{1-v_{s}^{2}}{E_{i}}$$

where *β* is a correction factor dependent on the indenter shape (1.034 for a Berkovich indenter). *Er* is the reduced modulus of specimens, *E_i_* and *E_s_* are the respective elastic modulus of the indenter and sample, and the *V_i_* and *V_s_* are the Poisson’s ratios of the specimens and indenter, respectively [35,36].

Additionally, the indentation Schmid factor, *S*, under Hertzian contact is presented as the ratio of the maximum resolved shear stress (among all possible slip systems) to the maximum contact pressure, defined as follows:

(6) $$S=\frac{1}{P_{0}}{Max}_{\left(v\right)x,y,z}\tau_{rss}^{\left(R\right)}(x,y,z)$$

where *S* is the Schmid factor; *P*_0_ is the maximum contact pressure in the contact area; $\tau_{rss}^{\left(R\right)}$ is the resolved shear stress on the *α*th slip system, with *α* running from 1 to the total number of slip systems; and *x*, *y*, and *z* are the displacement fields in the half-space solid under a unit point force vector applied at the coordinate origin.

## 3. Results

### 3.1. Microstructure Evolution

The actual chemical compositions of Ti-6Al-4V-*x*Fe alloys were measured using PS-6 (New York, NY, USA) inductively coupled plasma atomic emission spectrometry. The results are listed in Table 1, and they are similar with the nominal composition. Since the Fe content is almost the only difference between these alloys, the changes in the microstructure and mechanical properties are caused by the Fe additions.

**Figure 1:**
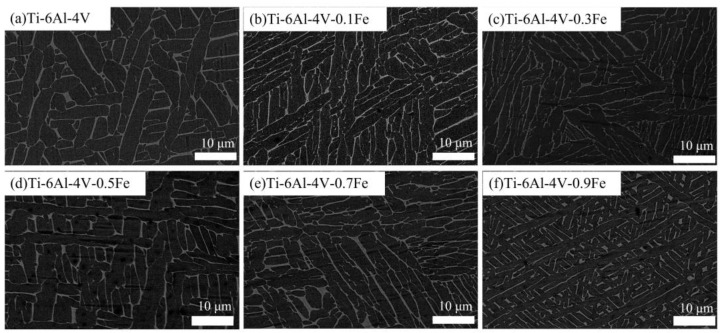
The microstructures of the Ti-6Al-4V-*x*Fe alloys: (**a**) Ti-6Al-4V; (**b**) Ti-6Al-4V-0.1Fe; (**c**) Ti-6Al-4V-0.3Fe; (**d**) Ti-6Al-4V-0.5Fe; (**e**) Ti-6Al-4V-0.7Fe; and (**f**) Ti-6Al-4V-0.9Fe.

Figure 1 demonstrates the micrographs of the investigated Ti-6Al-4V-*x*Fe alloys. Due to the SEM image analysis, the typical as-cast microstructure can be found. It consists of a lamellar α phase and matrix β-phase, which alternate side by side within the prior β grains and form colonies with the same orientation. The dark layer represents the α phase, and the white β layer represents the β-phase. The lamellar α + β microstructure was introduced into the previous β grains due to the solid phase transformation from β to α.

**Figure 2:**
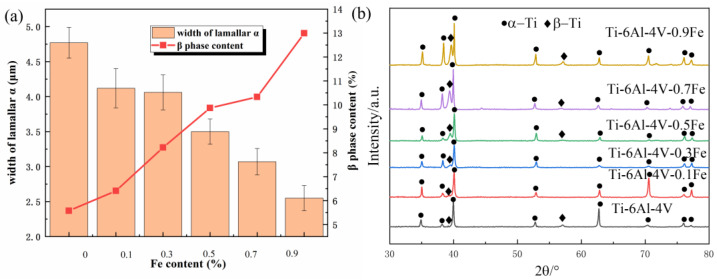
The width of lamellar α and the phase contents of different Ti-6Al-4V-*x*Fe alloys (**a**) and the XRD pattern of the alloy obtained by different methods (**b**).

According to the SEM images of these alloys, the data of the lamellar α width and β-phase content were obtained using Image Pro Plus. As presented in Figure 2, the Fe content shows an obvious influence on the microstructure features of the Ti-6Al-4V-*x*Fe alloys. The β-phase content increased from 5.8% to 13.0%, and the α phase content decreased from 94.2% to 87% with the increasing Fe content. This is due to the fact that Fe is considered to be the strongest β-stabilizing element, and Fe addition improves the stability of the β-phase and widens the homogeneity region of the β-phase to a lower temperature range.

The width of the lamellar α also shows a decreasing trend. Figure 2a summarizes the variation in microstructural features in relation to Fe addition. The average lamellar α width of Ti-6Al-4V is 4.75 μm, and it rapidly decreased to 2.5 μm for Ti-6Al-4V-0.9Fe. The error bars in the figure represent the statistical variation in the α phase width across different regions. With an increasing Fe content, a 47.37% decrease in the lamellar α size is observed, reflecting the refinement effect of Fe. A previous study [22] reported that the addition of Fe changed the growth limitation and nucleation of titanium alloys, greatly influenced the elemental distribution during solidification, and increased the nucleation rate during solidification. Also, Fe addition has a positive effect as it refines the nucleation inside the grains. Normally, the grain size affects the nucleation and growth of the solid α phase during solidification, and the phenomenon of mutual collisions between the growing α phase is more likely to occur in the refined β-grains [37]. These mechanisms interact together, leading to the refinement of lamellar α.

The XRD patterns are presented in Figure 2b; α-Ti and β-Ti can be recognized from the pattern, and the phase fraction of β-titanium varies depending on different amounts of Fe addition. The majority of the peaks in the XRD patterns are α phase, while the β-phase content is relatively low, which occurs only around 39° and 57° and is dominated at 39°. Comparing the peak intensities of different alloys, it can be found that the β-phase peak of Ti-6Al-4V is lower, but with the increase in the Fe content, the β-phase peak of the Ti-6Al-4V-xFe alloy near 39° increases gradually, and the β-phase peak of the alloy is the highest when the Fe content is 0.9 wt.%; additionally the intensity of the peaks associated with the β-phase in XRD becomes higher, which means that the content of the β-phase is on the high side. This is consistent with the observation in SEM that the content of the β-phase becomes higher as the Fe content increases.

### 3.2. Nano Micromechanical Properties

As one of the commonly used mechanics of material testing methods, nanoindentation can be carried out for the hardness and modulus of Ti-6Al-4V-*x*Fe alloys with different Fe contents. And the effect of the Fe content on the nanomechanical behavior, wear resistance, and plastic deformation properties was also investigated.

**Figure 3:**
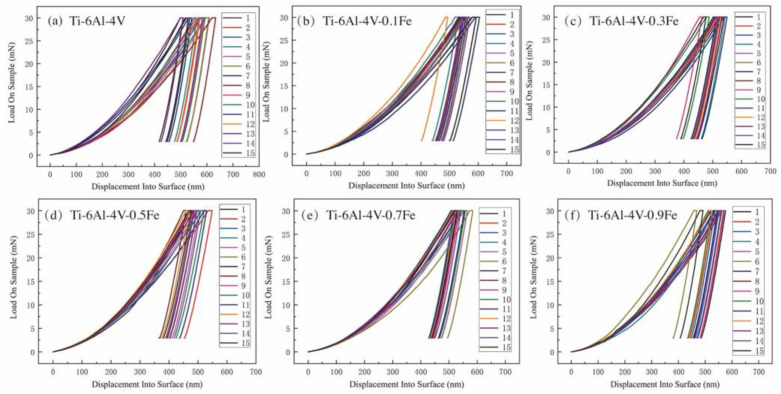
Load–displacement curves of Ti-6Al-4V-*x*Fe alloys: (**a**) Ti-6Al-4V; (**b**) Ti-6Al-4V-0.1Fe; (**c**) Ti-6Al-4V-0.3Fe; (**d**) Ti-6Al-4V-0.5Fe; (**e**) Ti-6Al-4V-0.7Fe; and (**f**) Ti-6Al-4V-0.9Fe.

The specific nanoindentation curves of Ti-6Al-4V-*x*Fe alloys are shown in Figure 3. The nanoindentation experiments were tested by the load control mode, and the maximum load is 30 mN. Each curve in the figure contains three parts, including the loading stage, the dwell time with the maximum loading, and the unloading stage. Firstly, in the loading stage, the indentation depth gradually increases. And then in the holding stage, the curve remains almost horizontal, resulting in a small amount of displacement. Finally, in the unloading stage, the load is gradually reduced to zero, and a small amount of pile-up occurs in the displacement, which is the recovery of the elastic deformation of the material, and the remaining displacement is caused by plastic deformation. The test is repeated 15 times for each alloy to ensure the accuracy of the experiment. And the slopes of the initial part of the unloading curve for the same alloy are almost equal, indicating that the results are accurate and repeatable.

**Figure 4:**
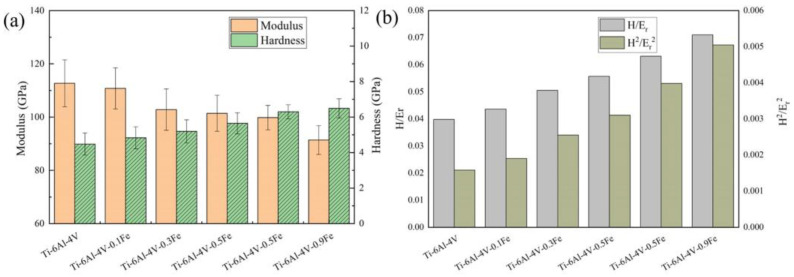
The variations in (**a**) the wear resistance (H/E) and (**b**) the resistance to plastic deformation (H3/E2) with different Fe contents.

The hardness and modulus data of the Ti-6Al-4V-*x*Fe alloys are listed in Figure 4a. It can be found that with the addition of Fe, the hardness and modulus have different trends. The error bars in the figure indicate the variation in hardness values from the repeated measurements. The modulus of Ti-6Al-4V is 112.69 GPa, and the hardness is 4.48 GPa. Compared with that, the modulus of Ti-6Al-4V-0.9Fe is 91.40 GPa, and the hardness is 6.49 GPa, respectively. These two parameters show different trends with different Fe contents. The microhardness increases by 44.87% with the rise in Fe content from 0 to 0.9 wt.% as Fe induces grain refinement and promotes a more homogeneous microstructure, positively influencing the hardness. Conversely, the relationship between the modulus and Fe content is inverse, as it decreases by 18.89% with 0.9 wt.% of Fe addition. This is attributed to the composition sensitivity of the elastic modulus of titanium alloys, and it has a correlation with the Mo equivalent [38]. Even trace changes in composition can lead to a substantial decrease in the modulus. The addition of Fe influences the distribution of Ti in the α and β-phases, thereby causing variations in the modulus.

Furthermore, the deformation properties of alloy materials can be expressed in terms of H/E_r_ and H^2^/E_r_^2^. The H/E_r_ ratio indicates the wear resistance of the alloy, which is related to the ability of the material to resist elastic strain damage [39]. The larger the H/E_r_ value, the better the wear resistance of the material. As shown in Figure 4b, the H/E_r_ ratio of Ti6Al4V-*x*Fe gradually increases from 0.040 to 0.071 as the Fe content increases, indicating that the Fe content improves the wear resistance of the alloy. Another parameter (H^2^/E_r_^2^) indicates the material’s plastic deformation resistance [40], and the larger the value of H^2^/E_r_^2^, the greater the plastic deformation resistance [41]. The trend in H^2^/E_r_^2^ is basically the same as the trend in the ratio of H/E_r_. It shows that Fe addition is beneficial to the wear resistance and plastic deformation resistance of the alloy.

There are two main factors that cause changes in mechanical properties. First of all, a titanium alloy is a kind of structure-sensitive alloy, and the change in the microstructure size leads to a change in the alloy hardness. According to previous studies, the smaller the grain size, the smaller the hardness and modulus [42,43].

Secondly, the main factor affecting the modulus is the phase content [44]. The addition of β-stabilizing elements in titanium alloys in various concentrations aid the formation of a β-phase. Prasad et al. [45] compared the differences between the transformed β and equiaxed α + β microstructures and found that β is more flexible and more sensitive to deformation compared to the α phase. Ruzic et al. [46] also concluded that different phase contents substantially influence the mechanical properties of titanium alloys, including the microhardness and modulus, and H_ω_ > H_α_ > H_β_ and E_ω_ > E_α_ > E_β_. The increase in the Fe content will cause an increase in the β-phase composition in the alloy, leading to a change in alloy hardness.

### 3.3. Microstructure Analysis after Nanoindentation

**Figure 5:**
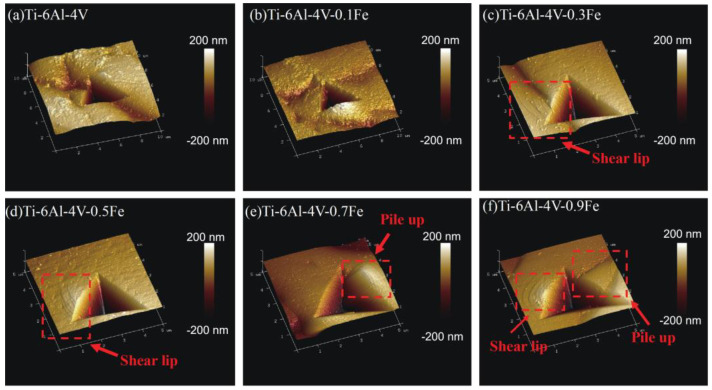
Three-dimensional topographical AFM images of different Ti-6Al-4V-*x*Fe alloys: (**a**) Ti-6Al-4V; (**b**) Ti-6Al-4V-0.1Fe; (**c**) Ti-6Al-4V-0.3Fe; (**d**) Ti-6Al-4V-0.5Fe; (**e**) Ti-6Al-4V-0.7Fe; and (**f**) Ti-6Al-4V-0.9Fe.

The atomic force microscopy (AFM) images of the indentation for Ti-6Al-4V-*x*Fe alloys are shown in Figure 5. Obviously, there is a difference between different amounts of Fe addition. It can be seen from Figure 5a that the vicinity of the indentation of Ti-6Al-4V is relatively flat, and the indentation shape is relatively regular, which is a triangle, with basically no pile-up. As the Fe content rises to 0.3 wt.%, a shear lip begins to appear around the indentation, which is characteristic of plastic deformation and represents the activation of more slip systems.

When the Fe content is higher than 0.5 wt.%, obvious pile-up starts to appear around the indentation. The locations of pile-up for different indentations vary from each other. This indicates that the formation and morphology of pile-ups are dependent on the composition. For each indentation, there are usually two hillocks located at two opposite sides of the Berkovich indenter, and one is usually higher and larger than the other. This reflects the localized plastic flow character of matrix material, which essentially relates to crystallographic slip systems. This also proves that pile-up behavior is dependent on the Fe content. It can be explained that Fe addition can improve the plasticity of the alloy and cause greater deformation of the structure around the indentation.

**Figure 6:**
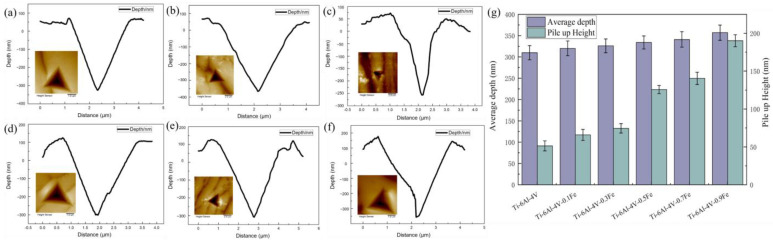
Cross-sectional analysis of different indentations of Ti-6Al-4V-*x*Fe alloys: (**a**) Ti-6Al-4V; (**b**) Ti-6Al-4V-0.1Fe; (**c**) Ti-6Al-4V-0.3Fe; (**d**) Ti-6Al-4V-0.5Fe; (**e**) Ti-6Al-4V-0.7Fe; and (**f**) Ti-6Al-4V-0.9Fe. (**g**) Average depths and pile-up heights of Ti-6Al-4V-*x*Fe alloys.

The depression depth and pile-up heights of the cross sections of different indentations can also reflect the plasticity of the alloy, as shown in Figure 6. The error bars in the figure represent the range in variation at different compression depths. It can be seen that as the Fe content increases, the depth of the recesses of the alloy increases, and the pile-up height around the indentation also increases. When the Fe content increases from 0 to 0.9, the pile-up height increases from 50 μm to 150 μm. This proves that the increase in the Fe content is more conducive to the formation and accumulation of pile-ups, thereby improving the deformation properties of Ti-6Al-4V-*x*Fe alloys.

## 4. Discussion

Since the β-phase of the Ti-6Al-4V-*x*Fe alloys is the matrix phase with little content and a small size, nanoindentation experiments could not be performed on the pure β-phase. The indentation was mainly distributed on the pure α phase and α + β-phase in this time. Furthermore, in this study, in order to exclude the influence of phase boundaries, a subsequent analysis was performed only on a single α phase (HCP).

### 4.1. Relations Between Fe Content and Elastic Compliance Coefficients

**Figure 7:**
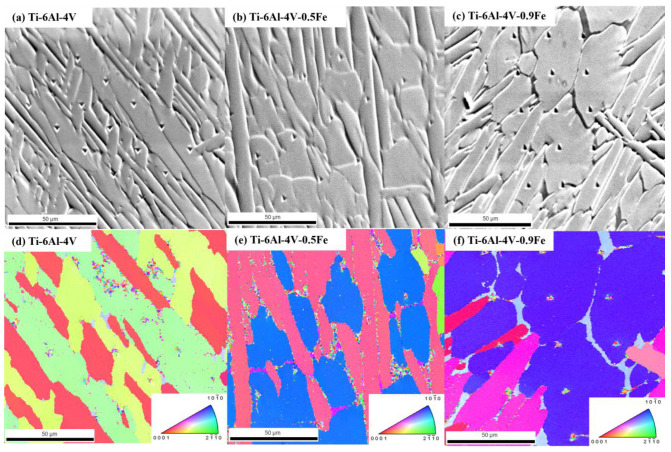
The nanoindentation microstructures of Ti-6Al-4V-*x*Fe alloys. (**a**–**c**) The SEM images of indentation marks. (**d**–**f**) The IPF images of Ti-6Al-4V-*x*Fe alloys. The color bar means different grain orientation of α phase.

The values of the four flexible elastic constants in the α phase of different alloys can be obtained from the elastic modulus, E. For further calculations, four indentations were selected from the 15 indentations of each alloy. Figure 7 was obtained using the electron backscattered diffraction technique, and the selected indentations were in the same phase and in the same grain orientation. Based on this, it is ensured that the Euler angles of these alloys are only influenced by the alloy composition, thus avoiding the influence of the alloy orientation.

**Figure 8:**
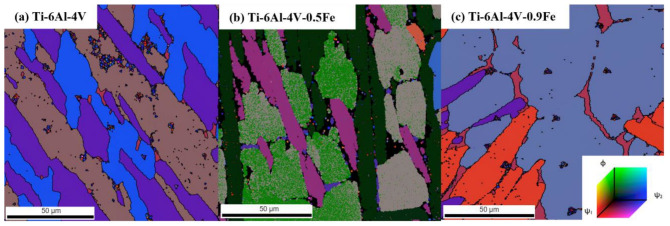
The Euler angle distribution maps of the Ti-6Al-4V-*x*Fe alloys: (**a**) Ti-6Al-4V; (**b**) Ti-6Al-4V-0.5Fe; (**c**) Ti-6Al-4V-0.9Fe. The color bar means different Euler angle direction.

The Euler angle distribution map of the Ti-6Al-4V-*x*Fe alloys was obtained by EBSD, as shown in Figure 8. It can be found that the Euler angle map is strongly associated with the Inverse Pole Figure (IPF) images. The specific Euler angle data near each indentation can be obtained from this map.

**Figure 9:**
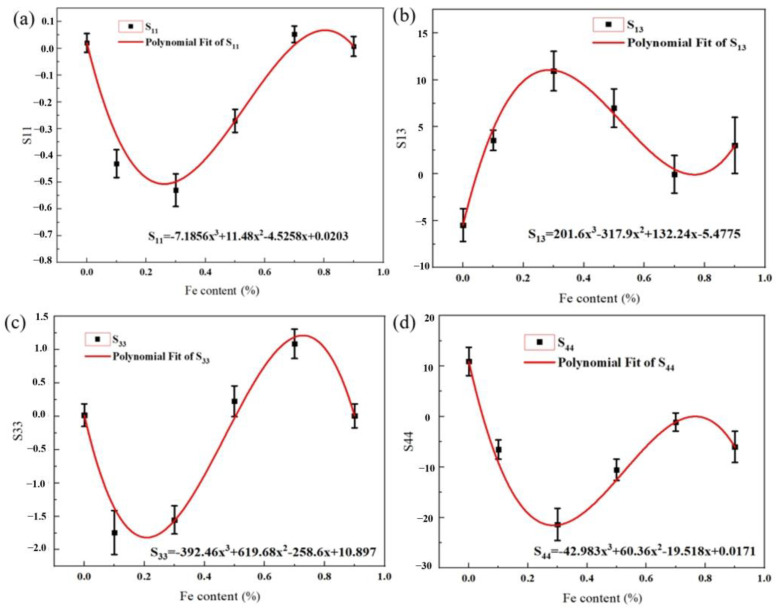
The relation between the Fe content and the elastic compliance coefficient: (**a**) S_11_; (**b**) S_13_; (**c**) S_33_; and (**d**) S_44_.

By extracting the Euler angles and moduli of these indentations, the elastic compliance coefficients of different alloys can be deduced, as shown in Table 2. And according to the variation in elastic constants of different alloys, the relationship between the elemental content of Fe and this constant can be obtained and expressed by polynomial fitting, as shown in Figure 9, where *x* represents the Fe content in wt.%. The error bars in the figure reflect the variation in elastic parameters across different alloys.

(7) $$\left\{\begin{array}{c} S_{11}=7.1856x^{3}+11.48x^{2}-4.5258x+0.0203\\ S_{13}=201.6x^{3}-317.9x^{2}+132.24x-5.4775\\ S_{33}=-392.46x^{3}+619.68x^{2}-258.6x+10.897\\ S_{44}=-42.983x^{3}+60.36x^{2}-19.518x+0.0171 \end{array}\right.$$

For the subsequent study, the equation can be used to derive the elastic compliance coefficients for different amounts of Fe addition, and we can thus calculate the elastic modulus of various alloys. Therefore, these equations could serve for the design of new materials in the future.

### 4.2. Schmid Factor Distribution and Slip System Activity

According to Balasubramanian et al. [47], common active slip systems in the hcp structure are the basal <a> slip (0001) <11$\bar{2}$0>, the prismatic <a> slip {10$\bar{1}$0} <11$\bar{2}$0>, the first-order pyramidal slip <c + a> {10$\bar{1}$1} <11$\bar{2}$3>, and the second- order pyramidal slip <c + a> {11$\bar{2}$2} <11$\bar{2}$3>. Among these slip systems, basal slip and prismatic slip are the main slip systems for the α phase in titanium. Since the addition of Fe may affect the activity of the slip system of these alloys, in order to further characterize the effect of Fe addition on the plasticity of titanium alloys, it is necessary to compare the slip systems and Schmid factors between different amounts of Fe addition.

Typically, the Schmid factor is often used to analyze the possible presence of activated slip systems. When the compression direction is given, the Schmid factor can be calculated from the EBSD data. Liu et al. [48] found that a higher Schmid factor is beneficial for the activity of the slip system.

**Figure 10:**
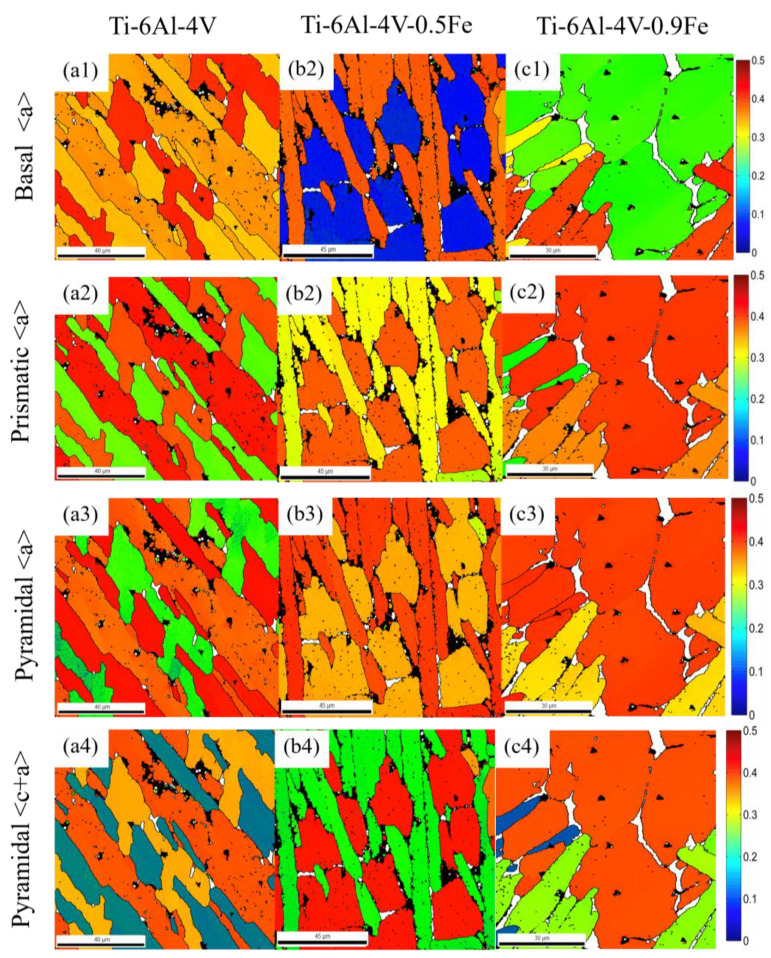
Schmid factor distribution maps and the average Schmid factor of different slip systems of Ti-6Al-4V-*x*Fe alloys: (**a**) Ti-6AL-4V; (**b**) Ti-6AL-4V-0.5Fe; and (**c**) Ti-6AL-4V-0.9Fe. (**a1**,**b1**,**c1**) is for Basal <a> slip system, (**a2**,**b2**,**c2**) is for Prismatic <a> slip system, (**a3**,**b3**,**c3**) is for Pyramidal <a> slip system, (**a4**,**b4**,**c4**) is for Pyramidal <c+a> slip system.

In order to analyze the variations in the Fe content on the Schmid factor, Figure 10 was constructed in which the distribution pattern of the Schmid factor for different alloys is statistically presented.

**Figure 11:**
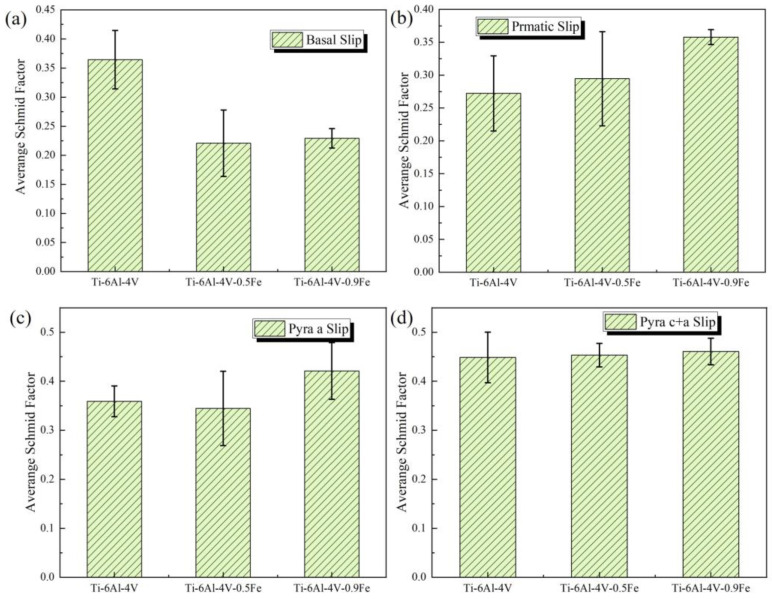
The average Schmid factor of different slip systems of different alloys: (**a**) basal <a>; (**b**) prismatic <a>; (**c**) pyramidal <a>; and (**d**) pyramidal <c + a>.

In these distribution maps, different colors represent the different deformation abilities of these alloys. As shown in the histogram distribution of Schmid factors in Figure 11, the grains change from hard orientation to soft orientation as the Schmid factor rises from 0 to 0.5 (the color changes from blue to red accordingly). It can be noticed that the Schmid factor of the selected indentation, which is located in a single α phase region, is barely influenced by the orientation.

In contrast, the Schmid factor varies a lot with different amounts of Fe addition. And the average Schmid factor was calculated for the different slip systems of each alloy, as shown in Table 3.

As shown in Figure 11, the Schmid factor becomes lower on the basal slip (0001) <11$\bar{2}$0> but higher on the prismatic slip {10$\bar{1}$0} <11$\bar{2}$0>, first pyramidal <c + a> slip {10$\bar{1}$1} <11$\bar{2}$3>, and second pyramidal <c + a> slip {11$\bar{2}$2} <11$\bar{2}$3> as the Fe content rises. The error bars in the figure represent the variation in Schmid factors across different slip systems for different alloys. Therefore, it can be concluded that Fe addition can improve the activity of the slip system on the prismatic and pyramidal slip systems of titanium alloys.

## 5. Conclusions

In summary, in order to analyze the effect of Fe addition on Ti-6Al-4V alloys, a comprehensive study of the microstructure evolution and nanomechanical properties of Ti-6Al-4V-*x*Fe alloys was carried out by nanoindentation, AFM, and EBSD analysis. The following conclusion can be made:

(1) Fe addition can lead to a significant refinement of the lamellar α phase. The mechanical properties of Ti-6Al-4V-*x*Fe alloys are improved with Fe addition. The microhardness increases by 44.87% with 0.9 wt.% of Fe addition. The modulus of Ti-6Al-4V-0.9Fe reaches 91.40 GPa, and it decreases by 18.89%, too. And Fe is also beneficial for wear resistance and plastic deformation resistance due to the increase in the H/E_r_ and H^2^/E_r_^2^ values.
(2) According to the morphology of this nanoindentation, the shear lips and pile-ups start to show up when the Fe content is higher than 0.3 wt%. When the Fe content rises from 0 to 0.9 wt.%, the height of the pile-ups rises from 50 μm to about 150 μm. This proves that Fe addition is beneficial for the formation and accumulation of pile-ups.
(3) Equations showing the relation between the Fe content and the elastic compliance coefficients were established. And they can be used to design and calculate the elastic modulus of new alloys with different amounts of Fe addition.
(4) The Schmid factors of the different slip systems of Ti-6Al-4V-*x*Fe alloys were studied. The alloys with higher Fe contents presented better slip activities in the prismatic slip {10$\bar{1}$1} <11$\bar{2}$3>, first pyramidal <c + a> slip {10$\bar{1}$1} <11$\bar{2}$3> and second pyramidal <c + a> slip {11$\bar{2}$2} <11$\bar{2}$3> systems.

## Figures and Tables

**Table 1:** The actual chemical compositions of Ti-6Al-4V-*x*Fe alloys.

Alloys	Ti	Al	V	Fe	C	N	O	H
Ti-6Al-4V	Bal.	5.98	4.10	0.03	0.014	0.003	0.083	0.0052
Ti-6Al-4V-0.1Fe	Bal.	5.92	4.05	0.13	0.015	0.006	0.080	0.0057
Ti-6Al-4V-0.3Fe	Bal.	5.99	4.09	0.33	0.012	0.004	0.084	0.0045
Ti-6Al-4V-0.5Fe	Bal.	5.95	4.07	0.52	0.014	0.003	0.076	0.0043
Ti-6Al-4V-0.7Fe	Bal.	5.92	4.02	0.73	0.016	0.005	0.081	0.0045
Ti-6Al-4V-0.9Fe	Bal.	5.99	4.10	0.91	0.013	0.004	0.083	0.0048

**Table 2:** The elastic compliance coefficients of Ti-6Al-4V-*x*Fe alloys.

Fe Content (%)	$S_{11}$	$S_{33}$	$S_{44}$	$S_{13}$
0	0.0203	0.0171	10.8974	−5.4775
0.1	−0.4306	−1.7423	−6.5351	3.5589
0.3	−0.5298	−1.5535	−21.3689	10.9309
0.5	−0.2709	0.2251	−10.5407	6.9905
0.7	0.0526	1.0872	−1.0952	−0.0729
0.9	0.0073	0.0071	−6.0096	3.0124

**Table 3:** The average Schmid factors of Ti-6Al-4V-*x*Fe alloys.

Fe Content (wt.%)	Basal	Prismatic	Pyra <a>	Pyra <c + a>
0	0.3644	0.2722	0.3591	0.4486
0.5	0.2208	0.2945	0.3446	0.4533
0.9	0.2292	0.3579	0.4210	0.4608

## Data Availability

The raw/processed data required to reproduce these findings cannot be shared at this time as the data are also part of an ongoing study.
